# CT-Based Analysis of Left Ventricular Hemodynamics Using Statistical Shape Modeling and Computational Fluid Dynamics

**DOI:** 10.3389/fcvm.2022.901902

**Published:** 2022-07-05

**Authors:** Leonid Goubergrits, Katharina Vellguth, Lukas Obermeier, Adriano Schlief, Lennart Tautz, Jan Bruening, Hans Lamecker, Angelika Szengel, Olena Nemchyna, Christoph Knosalla, Titus Kuehne, Natalia Solowjowa

**Affiliations:** ^1^Institute of Computer-Assisted Cardiovascular Medicine, Charité-Universitätsmedizin Berlin, Berlin, Germany; ^2^Einstein Center Digital Future, Berlin, Germany; ^3^Fraunhofer Institute for Digital Medicine MEVIS, Bremen, Germany; ^4^1000shapes, Berlin, Germany; ^5^Department of Cardiothoracic and Vascular Surgery, German Heart Center Berlin, Berlin, Germany; ^6^German Centre for Cardiovascular Research (DZHK), Partner Site Berlin, Berlin, Germany; ^7^Charité-Universitätsmedizin Berlin, Corporate Member of Freie Universität Berlin, Humboldt-Universität zu Berlin and Berlin Institute of Health, Berlin, Germany

**Keywords:** cardiac computed tomography, intraventricular hemodynamics, statistical shape modeling, fluid-structure interaction, computational fluid dynamics, left ventricle aneurysms, mitral regurgitation

## Abstract

**Background:**

Cardiac computed tomography (CCT) based computational fluid dynamics (CFD) allows to assess intracardiac flow features, which are hypothesized as an early predictor for heart diseases and may support treatment decisions. However, the understanding of intracardiac flow is challenging due to high variability in heart shapes and contractility. Using statistical shape modeling (SSM) in combination with CFD facilitates an intracardiac flow analysis. The aim of this study is to prove the usability of a new approach to describe various cohorts.

**Materials and Methods:**

CCT data of 125 patients (mean age: 60.6 ± 10.0 years, 16.8% woman) were used to generate SSMs representing aneurysmatic and non-aneurysmatic left ventricles (LVs). Using SSMs, seven group-averaged LV shapes and contraction fields were generated: four representing patients with and without aneurysms and with mild or severe mitral regurgitation (MR), and three distinguishing aneurysmatic patients with true, intermediate aneurysms, and globally hypokinetic LVs. End-diastolic LV volumes of the groups varied between 258 and 347 ml, whereas ejection fractions varied between 21 and 26%. MR degrees varied from 1.0 to 2.5. Prescribed motion CFD was used to simulate intracardiac flow, which was analyzed regarding large-scale flow features, kinetic energy, washout, and pressure gradients.

**Results:**

SSMs of aneurysmatic and non-aneurysmatic LVs were generated. Differences in shapes and contractility were found in the first three shape modes. Ninety percent of the cumulative shape variance is described with approximately 30 modes. A comparison of hemodynamics between all groups found shape-, contractility- and MR-dependent differences. Disturbed blood washout in the apex region was found in the aneurysmatic cases. With increasing MR, the diastolic jet becomes less coherent, whereas energy dissipation increases by decreasing kinetic energy. The poorest blood washout was found for the globally hypokinetic group, whereas the weakest blood washout in the apex region was found for the true aneurysm group.

**Conclusion:**

The proposed CCT-based analysis of hemodynamics combining CFD with SSM seems promising to facilitate the analysis of intracardiac flow, thus increasing the value of CCT for diagnostic and treatment decisions. With further enhancement of the computational approach, the methodology has the potential to be embedded in clinical routine workflows and support clinicians.

## 1. Introduction

Disorders of intraventricular hemodynamics are proposed to serve as an early biomarker for diagnosis of heart diseases, as they are associated with progressive remodeling of the left ventricle (LV) toward heart failure (1). A broad range of parameters has been proposed to quantitatively as well as qualitatively analyze *in vivo* blood flow by means of visualization techniques for various imaging techniques (2). Echocardiography is clinically the most used imaging modality and has been employed for the investigation of a wide spectrum of cardiac pathologies (3, 4), guidance of interventional procedures (5) or assessing the success of treatments (6, 7). However, echocardiography depends on an exact geometric alignment and is highly dependent on the operator. 4D flow magnetic resonance imaging (MRI) provides a higher spatial resolution with time-averaged assessment of intracardiac flow. Intraventricular kinetic energy has therewith been investigated (8) and large-scale flow patterns as well as vortex behavior have been topic of research (6, 9, 10). Downsides of 4D flow MRI are long acquisition times and limitations with implantable devices. Cardiac computed tomography (CCT) has the highest spatial resolution (11) but does not allow to capture intracardiac flow quantities.

Despite progressive developments in imaging modalities in recent years, a profound intraventricular blood flow analysis has not yet translated into clinical workflows. One major reason is the insufficient spatial and temporal resolution of echocardiography and MRI combined with intolerably long acquisition times of detailed 4D flow imaging. Another crucial factor impeding this translation from research to clinic is, that as for today, no consensus has been found regarding hemodynamic parameters or features distinguishing normal from pathological cases. Since pathological cases appear with a huge variety of changes in anatomy and heart function, it is challenging to compare inter-individual differences on the one hand, and on the other hand to identify characteristics by which patients can be sorted in groups to compare inter-group differences.

To overcome the first issue of limited resolution of imaging modalities, various research groups have proposed to use image-based computational fluid dynamics (CFD) to gain information about ventricular flow fields in combination with decreased patient scan times. Detailed reviews on different modeling approaches in cardiovascular medicine are provided by Quarteroni et al. (12), Doost et al. (13), and Hirschhorn et al. (14). Such image-based CFD frameworks can also be employed to investigate post-operative outcomes after virtual treatment (5, 15, 16). Furthermore, CFD can complement cardiac computed tomography (CCT) by functional analysis of patient-specific intraventricular hemodynamics via so-called CCT-based CFD (17). Despite being time-consuming and demanding high computational resources, CCT-based CFD may be an alternative to 4D flow MRI due to a higher possible spatial resolution and a reduction of scan times (18).

A combination of CFD simulations with a representation of clinical image data by means of statistical shape models (SSMs) can help to improve several aspects of image-based CFD models of the LV, as recently demonstrated by Khalafvand et al. (19). First, the integration and automation of medical image data and segmentations into the pre-processing workflow can be improved since it allows for description of complex shapes in a reduced manner. Further, SSMs can be used to analyze characteristics of different individuals or patient groups, e.g., using hierarchical cluster analysis (20) and may thus be a tool to find correlations between SSM shape parameters and biomechanical risk score (21), hemodynamic parameters (22, 23) or cardiac electromechanics (24). Eventually, SSMs are also used to generate synthetic cases in order to train machine learning algorithms (21, 25) or to investigate simulated blood flow in representative shapes of specific groups of patients (26–28).

In this work, we complement our previously introduced workflow for the comprehensive Fluid-Structure-Interaction CFD simulations of the LV of an entire heart cycle (29) by a SSM representation of LV geometry and motion. A cohort of 125 CCT examinations of heart failure patients after myocardial infarction, partly combined with mitral regurgitation (MR), is used for an extensive statistical shape analysis and clustering into seven subcohorts. For each subcohort, a mean shape representing the respective pathological state is derived from SSMs and used in the image-based CFD framework. Consequences of myocardial infarction (scar formation, disturbed contractility, and a dilation of LVs causing MR and an increase of the LV shape sphericity) are likely to alter the intracardiac flow. Our focus was therefore to investigate the intraventricular hemodynamics of the seven mean cases, each representing a different pathological state, and to reveal differences between the groups.

## 2. Materials and Methods

**Figure 1:**
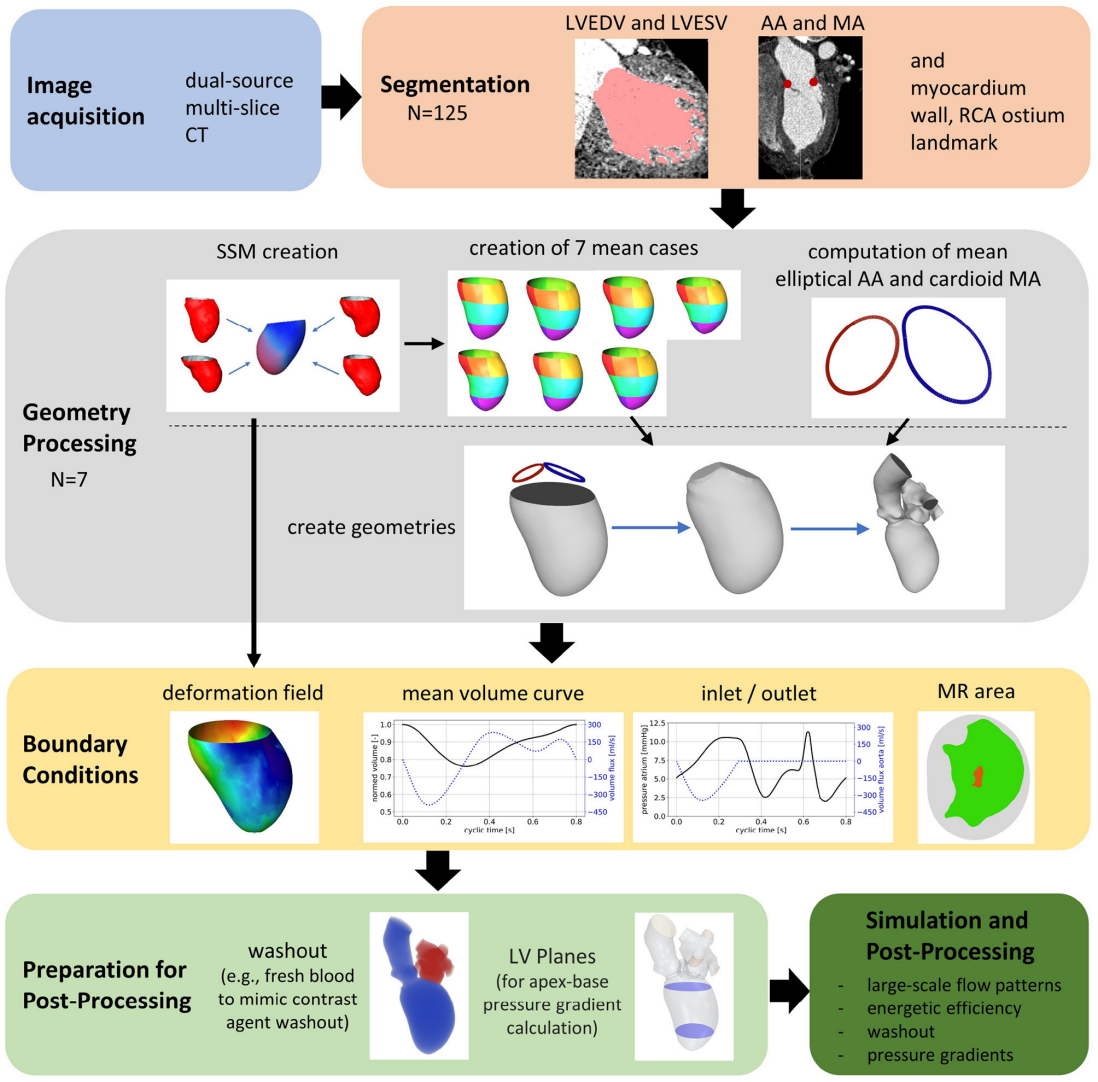
The central workflow of the study.

The general workflow of the numerical framework is displayed in Figure 1. Based on the acquired CCT images, left ventricular end-diastolic (LVEDV) and left ventricular end-systolic (LVESV) geometries as well as the end-diastolic myocardial wall, both annuli, and the ostium of the right coronary artery (RCA) are segmented. The data are used to create SSMs of the LV, including the ventricular contraction. Then, seven representative shapes of different groups of patients each representing a pathological state are generated using the SSMs. The generated cases are used as input of a recently developed computational framework for patient-specific simulations of the intraventricular flow. Finally, relevant hemodynamic parameters for the analysis of intracardiac flow are selected, calculated, and evaluated per case.

### 2.1. Study Cohort

Retrospective CCT data of heart failure patients after myocardial infarction (n=125, mean age of 60.6 ± 10.0 years, 16.8 % women) collected in the German Heart Center Berlin were used in this study. The patients are grouped into subcohorts in two ways: first, all 125 patients were subdivided into four groups, separating into patients without and with anterior LV aneurysm. Each of these two groups was further subdivided into patients with low MR (MR grade < II) and high MR (MR grade ≥ II). The different subcohorts are denominated via A (with 0 for non-aneurysmatic and 1 for aneurysmatic cases) and via M (with L indicating low MR grade < II and H indicating high MR grade ≥ II), resulting in the four subcohorts: A0ML, A0MH, A1ML, and A1MH. As second grouping, all cases with LV aneurysm were further subdivided into the three groups denoted via A1 [T = true aneurysms; I = intermediate aneurysms; HK = globally hypokinetic, which is also referred to as ischemic cardiomyopathy (30)]: A1T, A1I, A1HK. The definition of three LV aneurysm types was done according to Di Donato et al. (30). Briefly, true aneurysm cases are characterized by two changes in curvature in the LV geometry, intermediate aneurysms incorporate solely one such border, and globally hypokinetic cases have none. In five cases, the shape of the LV aneurysm was not evaluated. These cases were thus not classified and included into one of the subcohorts of different aneurysm types. The LV aneurysms were primarily diagnosed by echocardiography. The MR grade was quantified entirely by echocardiography. Table 1 summarizes clinical and demographic data of the seven subcohorts.

**Table 1:** Clinical and demographic data of the seven subcohorts.

**Parameters**	**A0ML**	**A0MH**	**A1ML**	**A1MH**	**A1T**	**A1I**	**A1HK**
Nr. of cases	40	32	35	18	10	22	16
Age (years)	60 ± 10.2	63 ± 9.2	59 ± 9.7	60 ± 11.2	59 ± 12.0	60 ± 10.0	58 ± 10.7
Sex (m/f)	34/6	28/4	27/8	15/3	6/4	17/5	14/2
BSA (m^2^)	1.99 ± 0.22	1.97 ± 0.16	1.91 ± 0.25	1.92 ± 0.20	1.91 ± 0.26	1.91 ± 0.22	1.89 ± 0.24
MR (grade)	1.0 [0.5]	2.5 [0.5]	1.0 [0.5]	2.25 [1.0]	1.0 [1.0]	1.0 [2.0]	1.25 [1.5]
NYHA class	III [0.5]	III [0.0]	III [0.0]	III [0.0]	III [0.0]	III [0.0]	III [0.25]
RV (ml)	7.8	29.8	8.1	25.8	11.9	15.8	10.3

*Values are shown as median [interquartile range] for both cohorts if a parameter is not normally distributed. Otherwise, values are displayed as mean ± standard deviation. The Shapiro-Wilk test was used to test normality. The Dubois formula (31) was used to calculate the BSA. LV, left ventricle; BSA, body surface area; MR, mitral regurgitation; NYHA, New York Heart Association; RV, regurgitation volume*.

### 2.2. Computed Tomography

CCT examinations were performed using a dual-source multi-slice spiral computed tomography scanner (Somatom Definition Flash, Siemens Healthcare GmbH, Erlangen, Germany). A spiral modus using retrospective electrocardiogram-gating was used to reduce motion artifacts from the heart. A tube voltage of 100 kV and an individually adapted tube current were applied to retrieve a multiphase data set resolving the heart cycle by 10 phases. This allows to assess LVEDV and LVESV. For image reconstruction, a standard soft-tissue convolution kernel and a dedicated noise reduction software were employed. Spatial resolution of the CCT images varied in the range of (0.390–0.648 mm) × (0.390–0.648 mm) for in-plane resolution and (0.5–1.85 mm) for the slice thickness. Temporal resolution varied between 70 and 140 ms, depending on the patient's heart rate.

### 2.3. Segmentation

**Figure 2:**
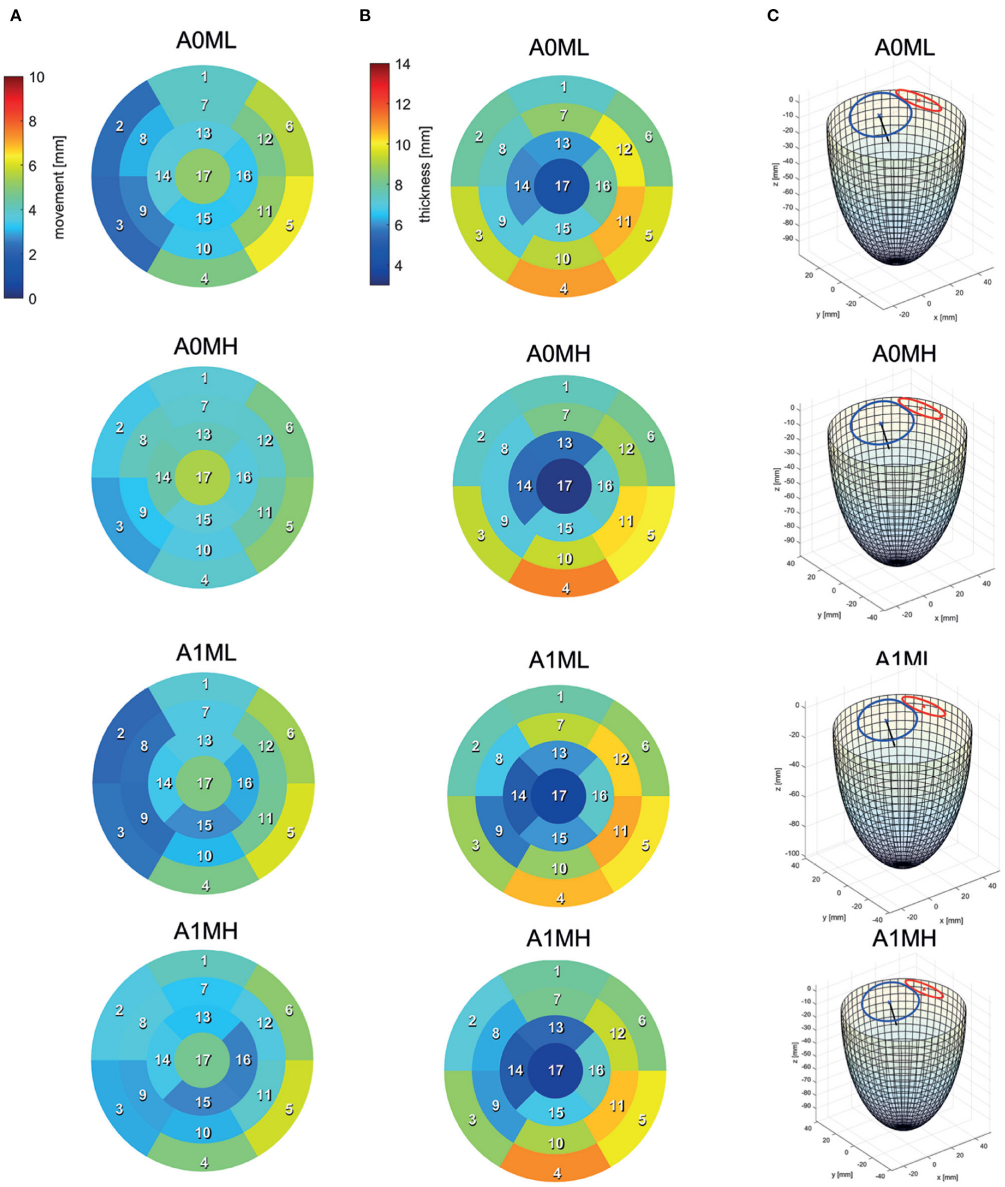
17-segment representations of **(A)** the myocardial wall movement (averaged wall distance from LVEDV to LVESV per patch) and **(B)** the myocardial wall thickness (segmented for the LVEDV) as well as wire-frame representations **(C)** of the end-diastolic LV for four subcohorts representing the whole study cohort.

**Figure 3:**
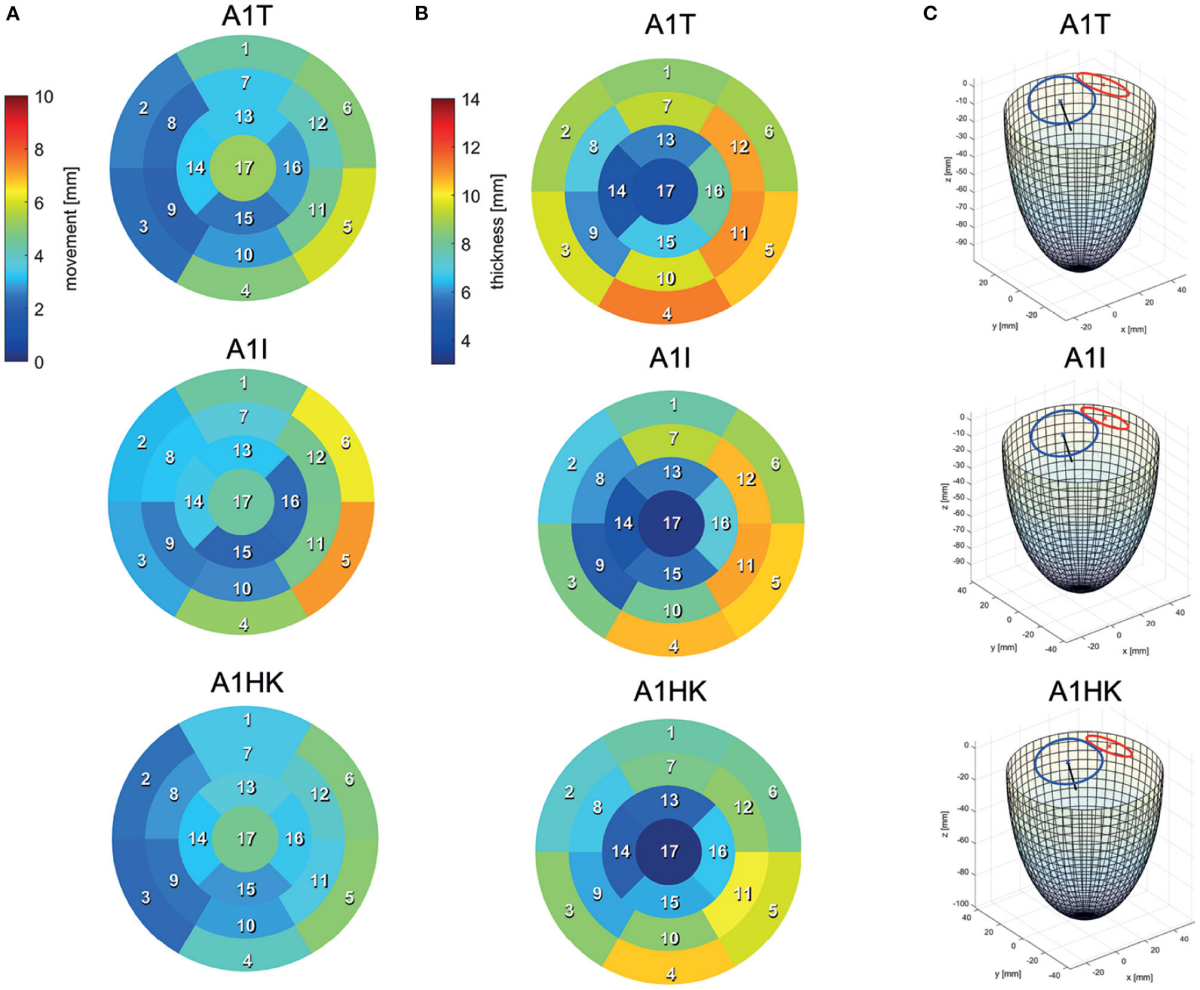
17-segment representations of **(A)** the myocardial wall movement (averaged wall distance from LVEDV to LVESV per patch) and **(B)** the myocardial wall thickness (segmented for the LVEDV) as well as wire-frame representations **(C)** of the end-diastolic LV for three subcohorts representing cases with different LV aneurysm types.

Segmentations are required to generate the SSMs of the LV. The segmentations were carried out using a recently developed in-house tool based on the MeVisLab platform (32) as described in detail by Tautz et al. (33). Briefly summarized, the LV in end-diastolic and end-systolic state is segmented semi-automatically building on an adaptive 3D region growing approach and contextual information of the heart topology. Where necessary, e.g., due to artifacts caused by metallic implants or uneven distribution of contrast agent, manual corrections were made. The segmented LV in end-diastolic state is used to manually segment the end-diastolic myocardial wall. Subsequently, both annuli were segmented by rotating 18 planes around the valvular axis, which was manually defined by setting two landmarks defining the LV apex and the respective center of the valve orifice. The annuli landmarks are used to interpolate the aortic annulus to an ellipse and the mitral annulus to a cardioid, which were used to define major geometric parameters of the annuli. As last, a landmark is set manually to define the RCA ostium. It is used for the registration of the segmented LVs, which is necessary to generate the SSM, which requires a similar orientation of input data. Furthermore, this allows a 17-segment analysis according to Cerqueira et al. (34), as well as visualizations of the myocardial wall thickness and wall movement during contraction. Figure 2 shows the 17-segment visualizations of the myocardial wall movement and wall thickness as well as wire-frame representations of the representative LVs for four subcohorts (which were defined based on MR and presence of an aneurysm), subdividing the whole study cohort of 125 cases. Figure 3 shows three subcohorts, subdividing the entire cohort into different aneurysm types. The segmentations were saved as DICOM files and used to generate triangulated surfaces required for the generation of the SSM and the measurement of the key geometric parameters: LVEDV, LVESV, LV sphericity index calculated according to (35), stroke volume (SV), ejection fraction (EF), and the areas of both annuli. Table 2 summarizes the averaged geometric parameters for all seven subcohorts. Two SSMs were generated to separate cases with and without LV aneurysms.

**Table 2:** Averaged geometric parameters for the seven investigated subcohorts.

**Parameters**	**A0ML**	**A0MH**	**A1ML**	**A1MH**	**A1T**	**A1I**	**A1HK**
LVEDV (ml)	275 [108]	304 [126]	313 [113]	279 [220]	222 [96]	324 [149]	324 [182]
LVESV (ml)	200 [102]	223 [97]	218 [98]	214 [187]	180 [74]	250 [127]	250 [166]
EF (%)	26.3 ± 7.8	25.3 ± 6.0	23.9 ± 6.7	23.4 ± 6.5	24.9 ± 6.9	24.1 ± 7.2	21.5 ± 5.2
SI	0.79 ± 0.16	0.87 ± 0.20	0.80 ± 0.20	0.88 ± 0.17	0.70 ± 0.14	0.88 ± 0.21	0.89 ± 0.15
MWT (mm)	8.3 ± 1.67	8.1 ± 1.37	8.0 ± 1.58	7.9 ± 1.04	8.5 ± 1.94	7.8 ± 1.39	7.7 ± 0.89
WM (mm)	3.86 ± 1.18	3.88 ± 1.13	3.60 ± 1.13	3.64 ± 1.24	3.51 ± 1.26	3.82 ± 1.01	3.36 ± 1.18
AVAA (cm^2^)	5.7 ± 1.05	5.9 ± 0.93	5.5 ± 0.99	5.6 ± 0.92	5.6 ± 1.22	5.7 ± 0.85	5.4 ± 1.02
MVAA (cm^2^)	10.2 ± 2.18	11.4 ± 2.13	9.9 ± 2.23	12.0 ± 2.90	9.0 ± 2.21	10.7 ± 2.50	11.2 ± 2.97

*Values are shown as median [interquartile range] for both cohorts if a parameter is not normally distributed. Otherwise, values are displayed as mean ± standard deviation. The Shapiro-Wilk test was used to test normality. LVEDV, left ventricular end-diastolic volume; LVESV, left ventricular end-systolic volume; EF, ejection fraction; SI, end-diastolic sphericity index; MWT, end-diastolic mean myocardial wall thickness; WM, mean wall movement; AVAA, end-diastolic aortic valve annulus area; MVAA, end-diastolic mitral valve annulus area*.

### 2.4. Statistical Shape Model

Prior to the actual SSM computation of the LVs, the segmentations need to go through a pre-processing step that subdivides the surfaces into areas, so-called patches. These patches are projected on a circular disc, so-called reference. Subsequently, a point correspondence can be computed between each individual surface geometry and the reference. In this SSM, we decided to design the patches according to the 17-segment heart model (34). This way, the patch-wise information can be later used for further analysis according to clinical routine workflows. To define the borders on the surface, the segmented landmarks are used to define separating planes, as follows. The plane defined through the RCA and apex is used to define all vertical borders of the 17 segments by rotating. A plane which is fitted to the annuli landmarks is used to cut the LV open right below the annuli landmarks. The plane is then displaced horizontally to 1/3 and 2/3 of the LV's height to add the horizontal borders. Apart from the end-diastolic surface information, a displacement field between end-diastolic and end-systolic surfaces is created by computing the closest points between the two surfaces. The displacement field can be used to recreate the end-systolic surface by displacing the end-diastolic vertices along the field while also preserving the patch information from the end-diastolic surface. Additionally, the surface distance is computed between the end-diastolic and the myocardium to store the myocardial thickness in a scalar field, which can be displayed as a color map on the end-diastolic surface. Our SSM approach is able to combine the end-diastolic surface, the displacement field to the end-systolic surface, the vector field of the myocardial thickness as well as the RCA landmark in one single model and is computed as follows. First, all training data is aligned to the first input training case. A data matrix with rows for features and n columns containing the training cases, is constructed. Most of the features are related to the m vertices of the end-diastolic surface and are encoded in the data matrix as shown in Equation 1.


(1) $$\begin{array}{r} M=\left(\begin{array}{cccc} s_{1,1} & s_{1,2} & \cdots & s_{1,n}\\ s_{2,1} & s_{2,2} & \cdots & s_{2,n}\\ \vdots & \vdots & \ddots & \vdots \\ s_{3*m,1} & s_{3*m,2} & \cdots & s_{3*m,n}\\ d_{1,1} & d_{1,2} & \cdots & d_{1,n}\\ d_{2,1} & d_{2,2} & \cdots & d_{2,n}\\ \vdots & \vdots & \ddots & \vdots \\ d_{3*m,1} & d_{3*m,2} & \cdots & d_{3*m,n}\\ v_{1,1} & v_{1,2} & \cdots & v_{1,n}\\ v_{2,1} & v_{2,2} & \cdots & v_{2,n}\\ \vdots & \vdots & \ddots & \vdots \\ v_{m,1} & v_{m,2} & \cdots & v_{m,n}\\ p_{1,1} & p_{1,2} & \cdots & p_{1,n}\\ p_{2,1} & p_{2,2} & \cdots & p_{2,n}\\ p_{3,1} & p_{3,2} & \cdots & p_{3,n} \end{array}\right) \end{array}$$

Therein, s represents the surface vertex coordinates, d the elements of the displacement field vectors, v the scalar values of the myocardial thickness per vertex, and p the coordinates for the RCA landmark. Using principal component analysis (PCA), the covariance matrix can be computed. The SSM is then described according to Equation 2.


(2) $$\begin{array}{r} S_{i}=\bar{S}+X\cdot b_{i} \end{array}$$

Therein, $\bar{S}$ is the mean shape, X the eigenvector matrix of the covariance matrix, and *b*_*i*_ the shape parameters of the modes. The method is based on previous work as described in more detail by Lamecker et al. (36).

Making use of the described procedure, two SSMs are computed: the first one using all cases with LV aneurysm, the latter one considering only cases without LV aneurysm. These two SSMs are then evaluated, e.g., in terms of mode analysis: the first three modes are compared visually and the cumulative variance of modes is calculated in order to assess the dimensionality reduction. Furthermore, seven mean cases are computed based on the respective differentiation into subcohorts as pointed out in Section 2.1. Each mean case represents a different pathological configuration.

### 2.5. CFD Simulation Setup

The mean shapes of all seven subcohorts are used to set up the simulation. A detailed description of the workflow can be found in our recently published work (29). Average shapes are computed for the cardioid mitral annulus and the elliptical aortic annulus which are used to reconstruct the entire LVEDV via a Poisson Surface Reconstruction algorithm. Non-case-specific reconstructions of a left atrium (LA) and an aorta are attached at the respective annulus of all seven representative SSM-based LV shapes. The SSM-based vector field prescribing the LV endomyocardial surface motion is scaled by a time-dependent factor to obtain a grid velocity vector allowing the ventricular volume to follow a specified volume curve at given EF. A grid velocity vector field results, which is prescribed as motion in the STAR-CCM+ (version 2021.2.1, Siemens Industries Digital Software, Plano, TX, USA) simulation.

The volumetric change of the LVs in time is described by a mean volume curve which was obtained from another cohort. This mean volume curve is scaled to a heart rate of 75 bpm (0.8 s per cycle) and the respective EF per case. At all walls, no-slip boundary conditions (BCs) in terms of relative movement of wall to fluid are posed. At the pulmonary veins, a physiological pressure BC is applied, whereas at the aorta, the respective mass flow rate is set, taking into consideration the blood that regurgitates into the LA. The MR grade determines the regurgitation fractions by correlating regurgitation fractions of 15, 30, and 50% to MR grades of I, II, and III (37) and interpolating linearly in between. The resulting regurgitation volumes are shown in Table 1. The valves are positioned in the annuli planes in a 2D-planar modeling approach (38, 39). For the aortic valve (AV), an elliptical opening area and for the mitral valve (MV) a projected orifice area, taken from Schenkel et al. (38), are used. Valve opening and closing is modeled via a porous baffle interface. Modeling moving obstacles as e.g., heart valves via porous media theory enables to model a surface movement via a spatio-temporal adaption of permeability parameters, being computationally efficient and easy to implement (40). To mimic valve opening and closing, a pressure drop according to Darcy's law is impressed onto the fluid via the porous baffle interface, as shown in Equation 3 (41).


(3) $$\begin{array}{r} \text{Δ}p=-\rho \left(\alpha |v_{n}|+\beta \right)v_{n} \end{array}$$

Therein, Δ*p* is the induced pressure drop over the interface, *ρ* the fluid density, α the user-specified porous inertial resistance, *v_n_* the fluid velocity normal to the interface, and β the user-specified porous viscous resistance. By varying this pressure drop in time and space, smooth 2D valve openings can be realized. The AV is opened elliptically, keeping the aspect-ratio of the AV in opened state. For the MV, an intermediate state, also taken from Schenkel et al. (38), is applied. For the MV, orifice areas of 5.65 cm^2^ and for the AV orifice areas of 4 cm^2^ are chosen. Based on the studies by Leyh et al. (42), the AV opening and closing time intervals are set to 57 and 39 ms. For the MV, opening and closing times of 48 ms and 60 ms were chosen. The regurgitation areas on the MV are chosen such that reasonable pressure losses over the MV result. A detailed description of the BCs as well as their detailed motivation can be found in Obermeier et al. (29).

STAR-CCM+ is used to create a polyhedral volume mesh at a base size of 1 mm with a refinement to 0.25 mm in valve regions where the highest velocity gradients are expected. The number of cells results in approximately 650,000. To retrieve reasonable initial conditions, two cycles were computed in advance. As results in a previous study suggested, a sufficiently swung-in state can be assumed after two cycles for such dilated LVs with reduced EF (29). In the same study, mesh convergence was shown for the specified mesh dimensions. Blood is modeled as incompressible with a density of 1,050 kgm^−3^ and as non-Newtonian Fluid according to the Carreau-Yasuda model. The model is parameterized with an infinity shear rate of 0.0035 Pas, a zero shear rate of 0.16 Pas, a relaxation time of 8.2 s, a power constant of 0.2128 and a = 0.64 (43). An arbitrary Lagrangian-Eulerian discretization method from STAR-CCM+ is used to solve the 3D incompressible Navier-Stokes equations with moving mesh for unsteady flow with no body-forces and source terms (Equations 4 and 5) (41).


(4) $$\begin{array}{r} \frac{\partial }{\partial t}\underset{V}{\int }\rho \text{d}V+\underset{\text{Ω}}{\int }\rho \left(\text{v}-{\text{v}}_{g}\right)\cdot \text{n}\text{dΩ}=0 \end{array}$$

(5) $$\begin{array}{r} \frac{\partial }{\partial t}\underset{V}{\int }\rho \text{v}\text{d}V+\underset{\text{Ω}}{\int }\left[\rho \text{v}\left(\text{v}-{\text{v}}_{g}\right)+p\text{I}-\text{T}\right]\cdot \text{n}\text{dΩ}=0 \end{array}$$

Here, *t* denotes time, *V* the control volume, Ω is the boundary of the control volume, **n** is the outwardly directed vector normal to *d*Ω, **v** is velocity, **v**_g_ is the grid velocity, *p* is pressure, *ρ* is density, **I** is the unit tensor of second order, and **T** is the viscous stress tensor. A RANS k-omega SST turbulence model and an implicit second order time-stepping scheme are applied.

In comparison to our previous work (29), we further automated the pre-processing procedures and optimized the time-consuming numerical framework. Pre-processing now required 4–8 h, whereas the cyclic blood flow computation took 12–13 h on 4 nodes at 40 cores (Intel Skylake 6,148, 2.3 GHz) on the Emmy system of the North-German Supercomputing Alliance. Due to an adaption of the deformation scaling factor into an implicit formulation (considering previous time steps), the pre-processing step of deformation computation is now omitted.

### 2.6. Post-processing of the LV Hemodynamics

The hemodynamics analysis is based on pressure and velocity fields as well as a passive scalar to investigate the blood washout process. A passive scalar is a passive tracer that can be placed in the fluid and moves according to the velocity field, without influencing the fluid motion itself (only convective transport is considered, herein). Therewith, a transport equation is solved to track the passive scalar motion, bypassing the need to consider multi-phase flow. To analyze the washout, the blood domain is separated into old and new blood with identical rheological properties by using two passive scalars. This modeling approach enables to mimic the clinically used blood washout behavior analysis by injecting contrast agent. The passive scalar for the old blood is placed in the LV in the initial state at *t* = 0 s, as e.g., done by Grünwald et al. (44). It is being washed out in the subsequent considered eight cycles. The passive scalar for fresh blood is placed in the LA at the onset of diastole of the first cycle at *t* = 0.29 s and mimics a contrast agent washout.

For an energetic evaluation, beside the normalized kinetic energy (normalized with the ventricular volume) called specific kinetic energy (SKE), the dissipation function Φ is used to quantify energetic losses. It is computed via the double-dot product of the velocity gradient vector ∇**v** and the stress tensor σ (45, 46) as shown in Equation 6.


(6) $$\begin{array}{r} \text{Φ}=\text{σ}\cdot \cdot \nabla \text{v} \end{array}$$

For the investigation of intraventricular pressure gradients, two horizontal planes are positioned in the LV: one in basal and one in apical region. The intraventricular pressure gradient is then computed by the difference of the average pressure per plane.

## 3. Results

First, both SSMs as well as the seven mean geometries and contractions are analyzed. Subsequently, the hemodynamics are evaluated by means of large-scale flow patterns, energetic performance, blood washout behavior, and intraventricular pressure gradients. Therein, differences between the pathological states are emphasized by comparing two particular LV groups. The first group consists of cases A0ML, A0MH, A1ML and A1MH, separating cases with and without LV aneurysm as well as different degrees of MR (low and high). The second group is composed of cases with different characteristics of the LV aneurysm, i.e., A1MT, A1MI, and A1HK, all being accompanied by a medium MR grade.

### 3.1. Statistical Shape Model

The two developed SSMs aim to describe pathological LVs of patients with and without LV aneurysms, including a description of shape, deformation due to contraction, and myocardial wall thickness. Identification of principal modes allows for a reduction of complexity and dimensionality. Note, that the MR degree was not included into the SSM but is directly defined through the computational model via the MR area during systole.

**Figure 4:**
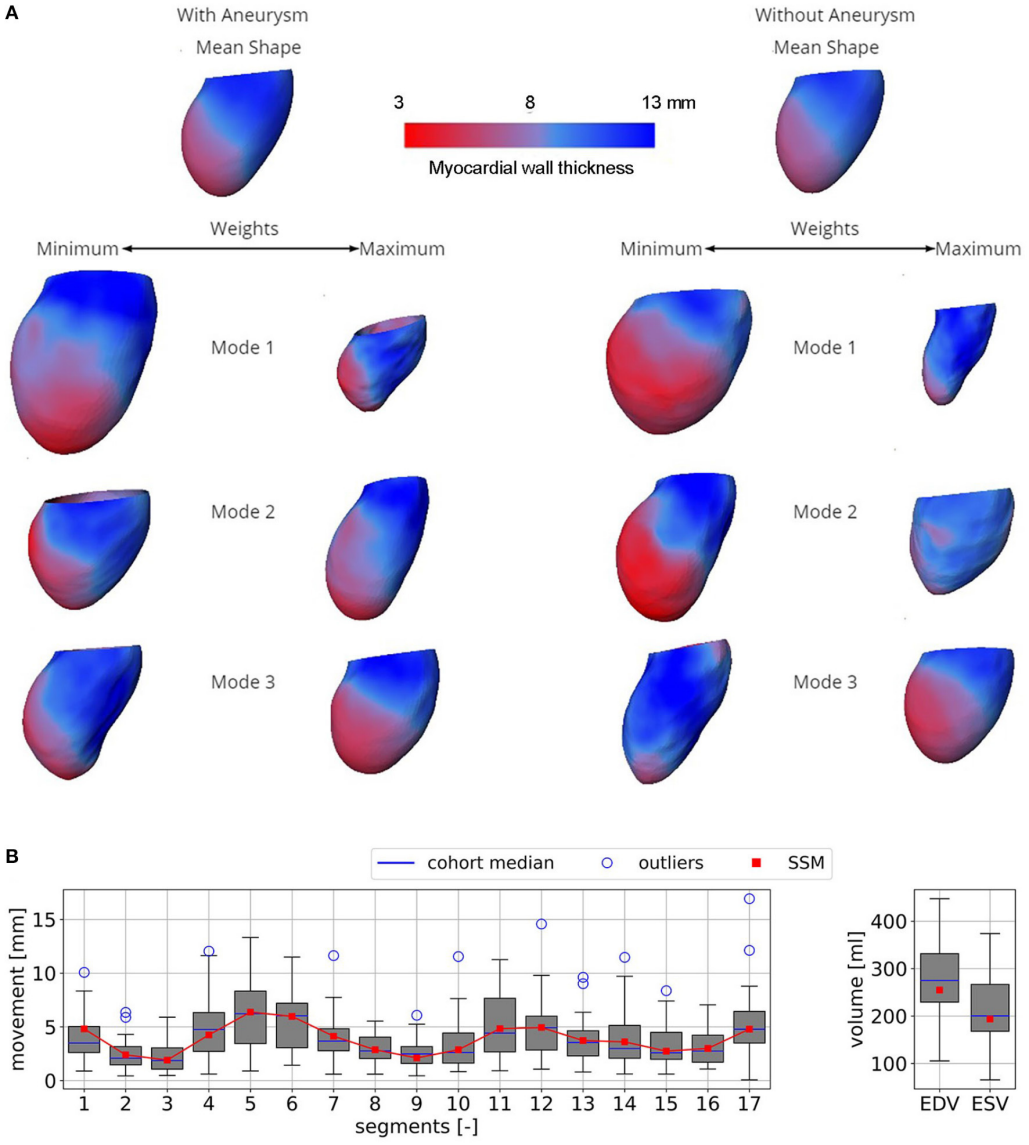
**(A)** Both SSMs of the LV with and without aneurysm represented by mean end-diastolic shapes colored by the myocardial wall thickness. Three first most descriptive shape modes, representing the variance of the models, are shown. When changing the weight of a mode, certain features of the model are altered as well. **(B)** Boxplots of the mean segment-wise movement (left) and boxplots of EDV and ESV (right) exemplary shown for case A0ML.

Figure 4A shows the resulting SSMs. Visually, both mean shapes are very similar. However, comparison of the first three modes and their variances by weights shows remarkable differences. Mode 1 of the non-aneurysmatic SSM reveals a higher sphericity by comparing minimum weights, whereas the maximum weight of the aneurysmatic shape clearly indicates the LV aneurysm: a bulge left from the apex associated with relatively low myocardial thickness can be observed. Mode 2 of both SSMs shows a variance in the shape sphericity of the LV with higher variability of the myocardial wall thickness in the non-aneurysmatic SSM in the apex region. The third modes are similar but show a higher variability for the myocardial wall thickness in the non-aneurysmatic SSM. In the process of dimension reduction by identifying principal modes, the aneurysmatic SSM requires 35 modes to include 95% of the cumulative variance, whereas the non-aneurysmatic SSM requires 38 modes.

**Figure 5:**
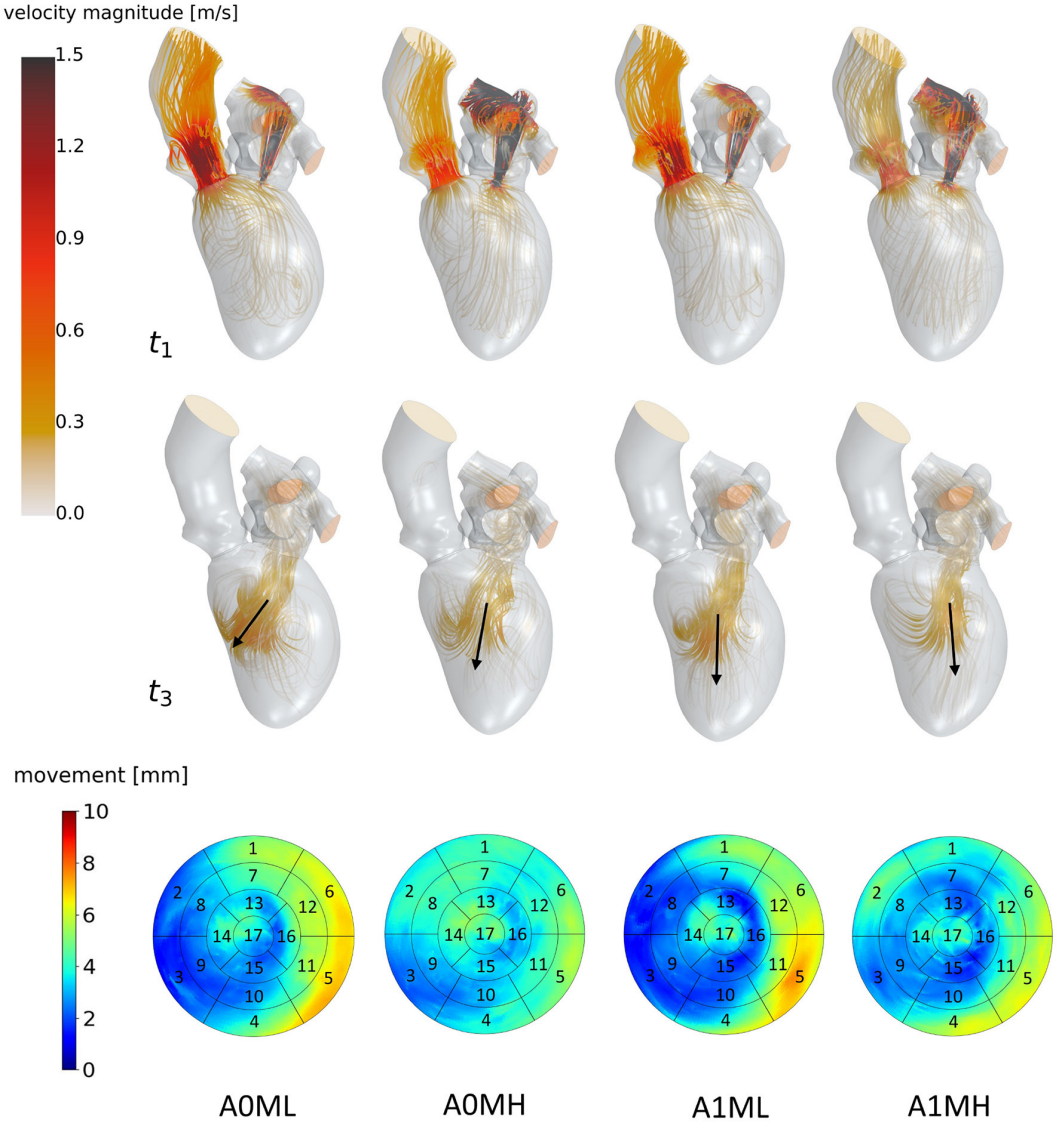
Streamlines of cases A0ML, A0MH, A1ML and A1MH from left to right at *t*_1_: peak systole (top) and *t*_3_: diastasis (mid) of the seventh cycle. At low velocity regions, streamlines are made transparent to underline the major flow features. Black arrows indicate the diastolic jet orientation. The bottom row shows the LV deformations at the surfaces. The black boxes and numbers display the regions corresponding to the 17-segment model.

**Figure 6:**
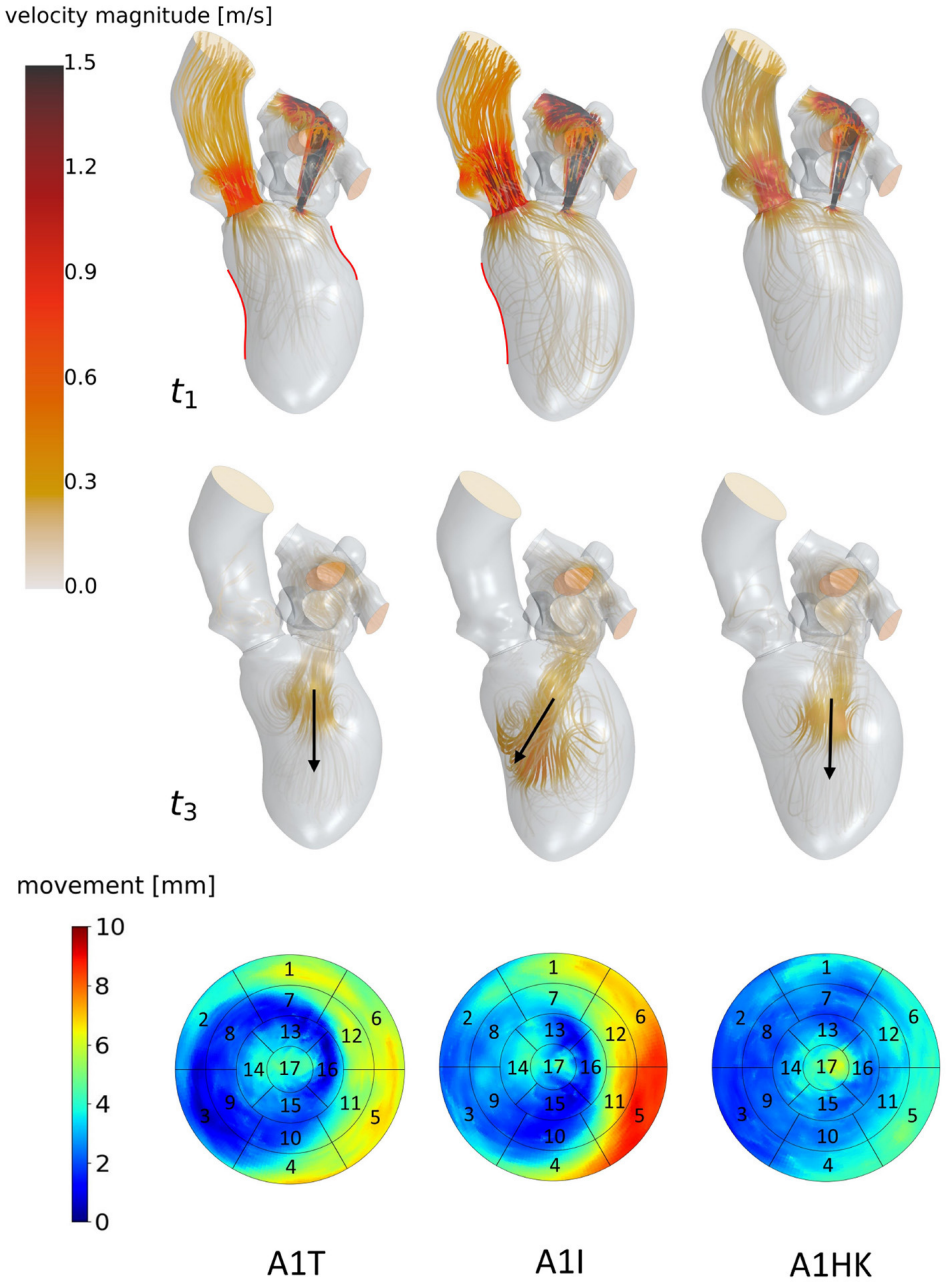
Streamlines of cases A1T, A1I and A1HK from left to right at *t*_1_: peak systole (top) and *t*_3_: diastasis (mid) of the seventh cycle. At low velocity regions, streamlines are made transparent and velocities above 1.5 m/s are clipped. Black arrows indicate the diastolic jet orientation. Red contours at *t*_1_ show changes in curvature to distinguish between different aneurysm types. The bottom row shows the LV deformation at the surfaces. The black boxes and numbers display the regions corresponding to the 17-segment model.

Figure 4B exemplary compares the 17-segment-wise wall movement during contraction as well as LVEDV and LVESV values of the A0ML cohort (40 cases) as a boxplot vs. the values of the representative mean shape of the cohort, created by the SSM. The values of the mean case are close to the median values of the cohort. Similar results are found for the other 6 representative mean cases. Comparing the deformations of the mean cases in Figures 5, 6 to the cohort-wise averaged ones as shown in Figures 2, 3 also reveals a good match of the myocardial wall movement.

The last point to address is to what extent the seven mean shapes represent the respective pathological state. Concerning the first group of cases (A0ML, A0MH, A1ML, and A1MH), less deformation can be seen in segments 13, 15, and 16 in the aneurysmatic cases A1ML and A1MH due to the anterior aneurysm (Figure 5 bottom). Furthermore, the non-aneurysmatic and high MR grade case reveals a more homogeneous myocardial wall deformation as respectively the aneurysmatic and low MR degree case. The three aneurysm types in cases A1T, A1I, and A1HK as defined by Di Donato et al. (30) can also be observed in the red marked regions in Figure 6 at *t*_1_. Case A1T shows two changes in curvature in systolic state, case A1I only one and case A1HK shows no change in curvature.

### 3.2. Large-Scale Flow Patterns

Figure 5 visualizes differences between major flow features of cases A0ML, A0MH, A1ML, and A1MH during peak systole (*t*_1_) and diastasis (*t*_3_). At peak systole, the majority of blood flows into the ascending aorta in all cases. The other portion regurgitates into the LA in form of a high velocity jet, impinging on the upper LA wall (Figure 5 *t*_1_). In the low MR grade cases, higher velocities appear in the left ventricular outflow tract (LVOT). The AV velocities at peak systole are between 0.69 and 1.03 m/s. The regurgitation jet velocities are between 3.84 and 4.38 m/s at pressure drops of 53–77 mmHg. During diastole, a blood jet enters the LVs from the LAs, causing the formation of commonly observed ring vortices. During diastasis, the jets become less coherent, especially in the cases with high MR. In cases A0MH, A1ML, and A1MH, the jets travel along the LV axis toward the apex. In case A0ML, it is directed at the lateral to anterior wall, where it impinges (Figure 5 *t*_3_). In these lateral and anterior regions in basal and mid LV regions, (i.e., segments 1, 5, 6, 7, 11, and 12), also a distinct LV movement is visible in case A0ML (Figure 5 bottom). In the other non-aneurysmatic case A0MH, a rather homogeneous contraction is present. In the aneurysmatic cases A1ML and A1MH, little movement is observed in the apical inferior and lateral region (i.e., segments 15 and 16) due to the development of aneurysms.

Figure 6 identifies differences in flow structures of cases A1T, A1I, and A1HK with different types of LV aneurysm and similar MR grades. A likewise systolic flow is observed in all three cases, consisting of an aortic outflow with velocities between 0.67 and 1.00 m/s as well as regurgitating jets at velocities from 3.88 to 4.44 m/s, resulting in MV pressure gradients of 66 to 73 mmHg (Figure 6 *t*_1_). Case A1I has a higher SV at comparable regurgitation volume, thus showing higher velocities in the aorta during systole as well as higher velocities of the diastolic jet (Figure 6 *t*_3_). The diastolic jets are accompanied by ring vortices. When reaching diastasis, the diastolic jet in case A1I traveled further and is directed toward the lateral to anterior wall, where it impinges. In cases A1T and A1HK, the jets move along the LV axis. Cases A1T and A1I show almost no movement in segments 15 and 16, while being characterized by a strong deformation in segments 5, 6, 11, and 12 in lateral and anterior regions (Figure 6 bottom). Case A1HK reveals a distinctively different deformation with the strongest contraction in apex region and less movement elsewhere.

### 3.3. Energetic Performance

**Figure 7:**
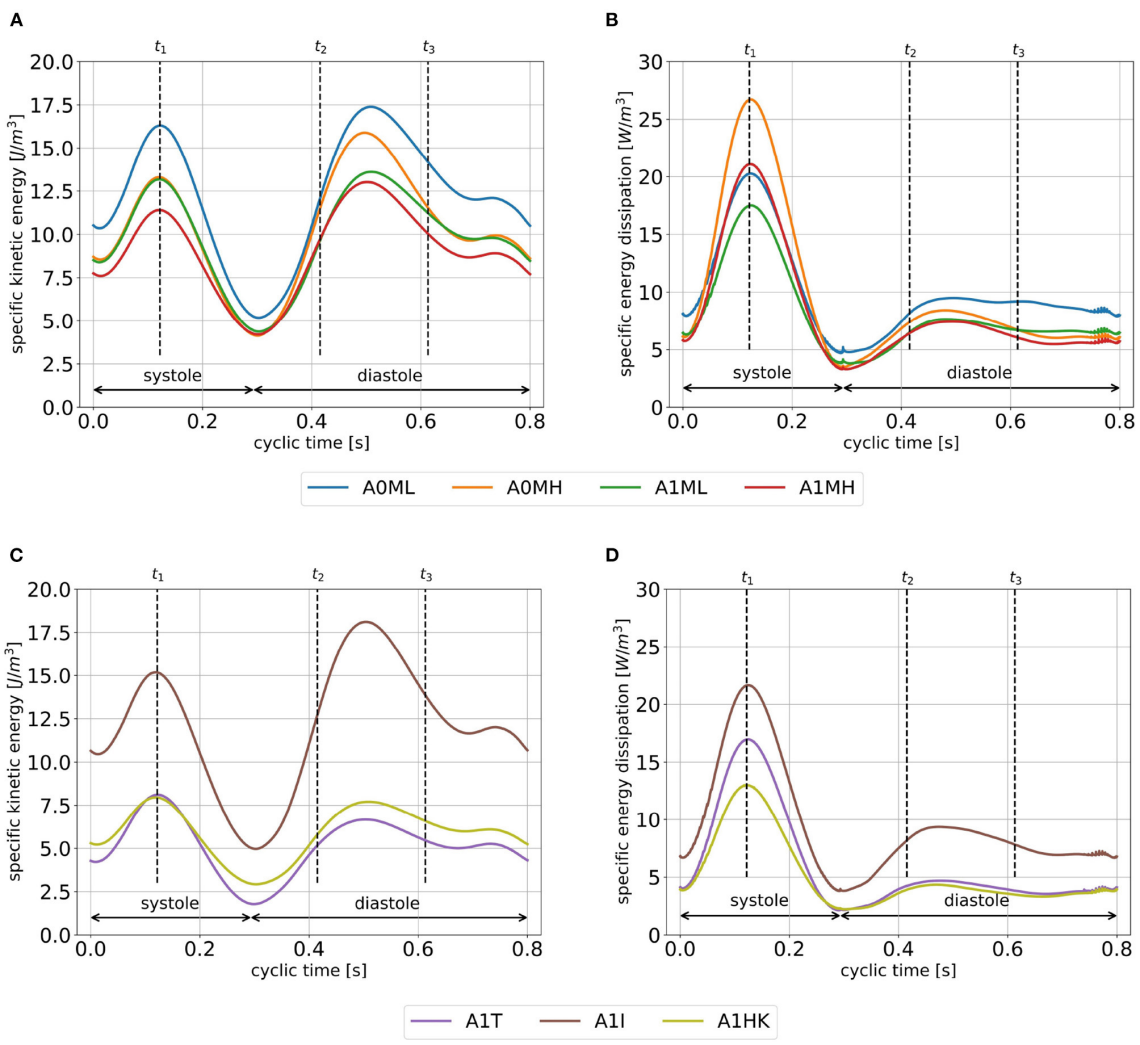
SKE **(A,C)** and SED **(B,D)** averaged over cycles one to seven. Time points *t*_1_, *t*_2_ and *t*_3_ mark peak systole, peak E-wave, and diastasis.

The specific kinetic energy (SKE) and specific energy dissipation (SED) take a qualitatively similar temporal course for all cases: the SKE has local maxima in peak systole, in the middle between peak E-wave and diastasis as well as during A-wave (Figures 7A,C). The SKE during systole is within the same order of magnitude as in diastole. Local minima can be observed as blood inside the LV decelerates and stagnates shortly after beginning of systole, at the end of the systolic phase, and in the middle of the A-wave deceleration phase. Comparing the four cases with and without LV aneurysms (Figure 7A), where SVs are similar, the cyclic mean of SKE reveals lower values in the aneurysmatic cases (A0ML: 12.2 J/mm^3^, A0MH: 10.3 J/mm^3^, A1ML: 9.8 J/mm^3^ and A1MH: 9.0 J/mm^3^). Furthermore, higher MR grades can be associated with lower SKE for both groups, with and without LV aneurysms. Comparing the three cases with different LV aneurysms (Figure 7C), case A1I with the largest SV (see Table 3) is characterized by a remarkable larger SKE if compared to A1I and A1HK. Regarding SED, a five-fold higher maximum in systole than in diastole is observed (Figures 7B,D). Furthermore, the SED during systole reveals inverse trends if compared with SKE: higher SED in the non-aneurysmatic cases as well as higher SED at higher MR. Comparing the three aneurysmatic cases, case A1I, which was associated with the highest SKE, is also associated with the highest SED. Finally, during systole there is a noteworthy difference in SED between A1T and A1HK, which show similar values for SKE.

**Table 3:** Intraventricular blood washout measures of the seven cases.

**Parameters**	**A0ML**	**A0MH**	**A1ML**	**A1MH**	**A1T**	**A1I**	**A1HK**
direct flow F.B. relative (%)	25.5	15.9	18.0	14.1	30.8	12.9	5.9
direct flow F.B. absolute (ml)	18.1	11.6	12.1	9.6	16.3	10.0	3.3
half-life F.B. (s)	2.60	2.65	3.27	3.33	2.46	2.61	4.87
half-life F.B. (cycles)	3.2	3.3	4.1	4.2	3.1	3.3	6.1
O.B. after 8 cycles (%)	11.52	10.17	13.87	15.02	19.00	10.04	21.47
SV (ml)	70.73	72.80	67.31	68.23	52.91	77.38	56.29

*The half-life marks the time when 50% of fresh blood are ejected. SV: stroke volume. F.B., fresh blood; O.B., old blood*.

### 3.4. Intraventricular Washout

**Figure 8:**
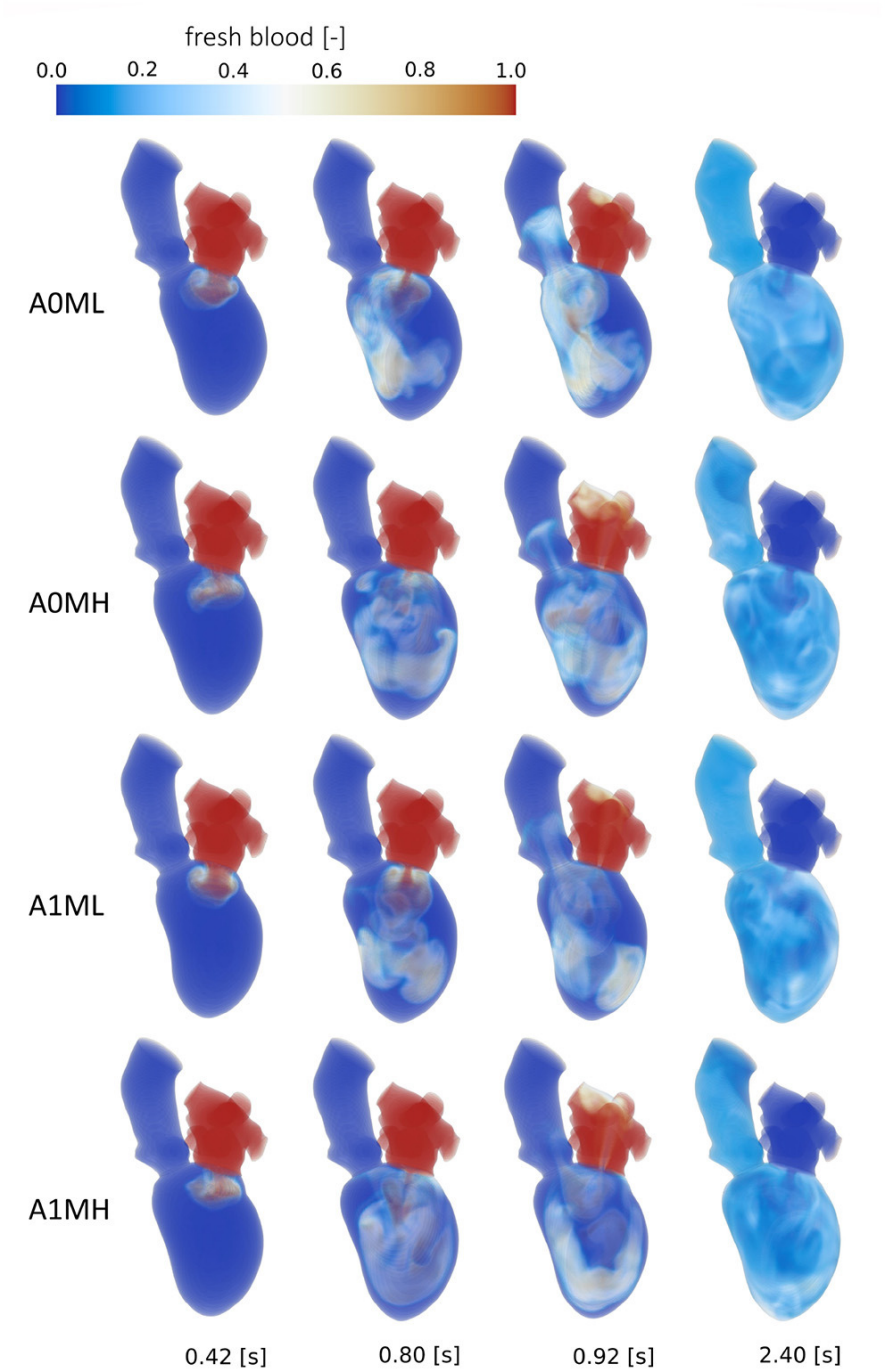
Washout of fresh blood of cases A0ML, A0MH, A1ML and A1MH from top to bottom at, from left to right, peak E-wave of the first cycle (0.42 s), end diastole of the first cycle (0.8 s), peak systole of the second cycle (0.92 s) and the end of the third cycle (2.4 s).

**Figure 9:**
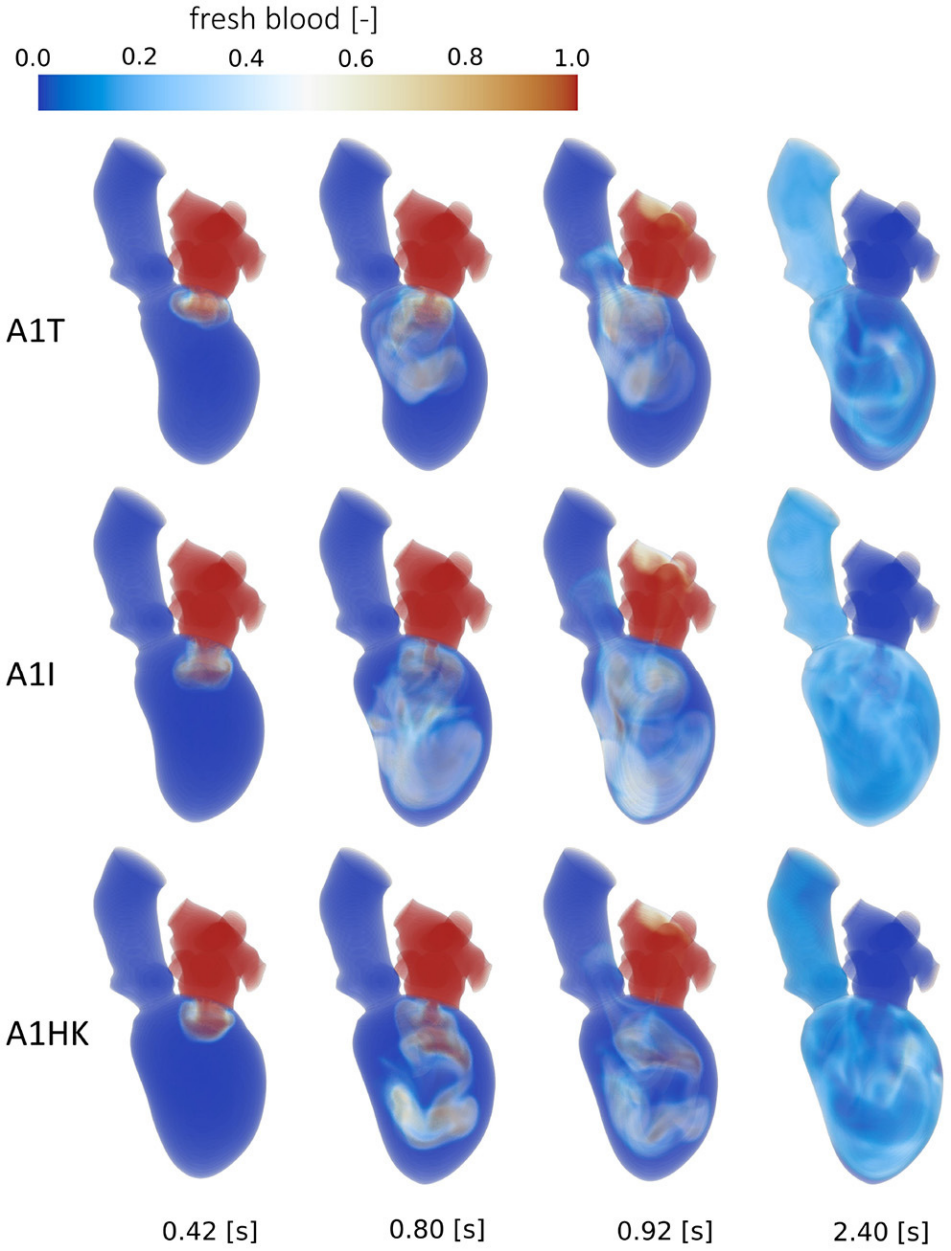
Washout of fresh blood of cases A1T, A1I and A1HK from top to bottom at, from left to right, peak E-wave of the first cycle (0.42 s), end diastole of the first cycle (0.8 s), peak systole of the second cycle (0.92 s) and the end of the third cycle (2.4 s).

The intraventricular blood washout is quantified by relative and absolute volumetric fractions of fresh and old blood (Table 3) and qualitatively analyzed via the washout of fresh blood (Figures 8, 9).

Comparison of the four aneurysmatic vs. non-aneurysmatic cases shows a reduced blood washout in the aneurysmatic cases, reaching the half-life of fresh blood approximately one heart cycle later (3 cycles vs. 4 cycles). Interestingly, the difference in the half-life between cases with low and high MR is relatively small, despite the lower direct flow rates in the high MR cases. After eight cycles, there is a larger percentage of old blood being still present in the aneurysmatic LVs. Figure 8 visualizes the blood washout behavior over a period of three heart cycles represented by four time steps: peak E-wave of the first cycle (0.42 s), end diastole of the first cycle (0.8 s), peak systole of the second cycle (0.92 s), and the end of the third cycle (2.4 s). The first and last represented time steps are very similar for all four cases. Time points at 0.8 s and 0.92 s appear to be most suitable for the visual differentiation between cases. At the end of the first cycle, the fresh blood did penetrate toward the apex in all cases (Figure 8 at 0.8 s). In case A0ML, the jet is directed toward the lateral to anterior wall. In the opposite regions (i.e., inferior and septal), a region with poor mixing appears, being still visible in peak systole of the second cycle (Figure 8 at 0.92 s). In the aneurysmatic cases A1ML and A1MH, regions with poor mixing can be observed at that time as well: inferior and septal near-wall region in the A1ML case and central LV region in the A1MH case. In case A0MH, the most homogeneous mixing seems to be present.

A comparison of the washout behavior between the three cases with different characteristics of the LV aneurysm (i.e., A1T, A1I, and A1HK) reveals the by far highest direct flow rate in case A1T. Yet, the half-lives of fresh blood are similar in cases A1T and A1I, whereas it is significantly longer in case A1HK. In terms of old blood washout, the analysis found that cases A1T and A1HK are related to the worst washout, still having 20 % of the old blood inside the LVs after eight cycles. In the qualitative washout analysis (Figure 9), a poor mixing of fresh blood especially at 0.8 s and 0.92 s in cases A1T and A1HK can be observed. In case A1T, the jet of fresh blood does not penetrate all the way to the apex and remains in the area below the AV, mixing poorly. In case A1HK, the jet reaches the apex, but rarely mixes with the rest of the blood. In both cases, unmixed regions are still visible at the end of the third cycle at 2.4 s.

### 3.5. Intraventricular Pressure Gradients

**Figure 10:**
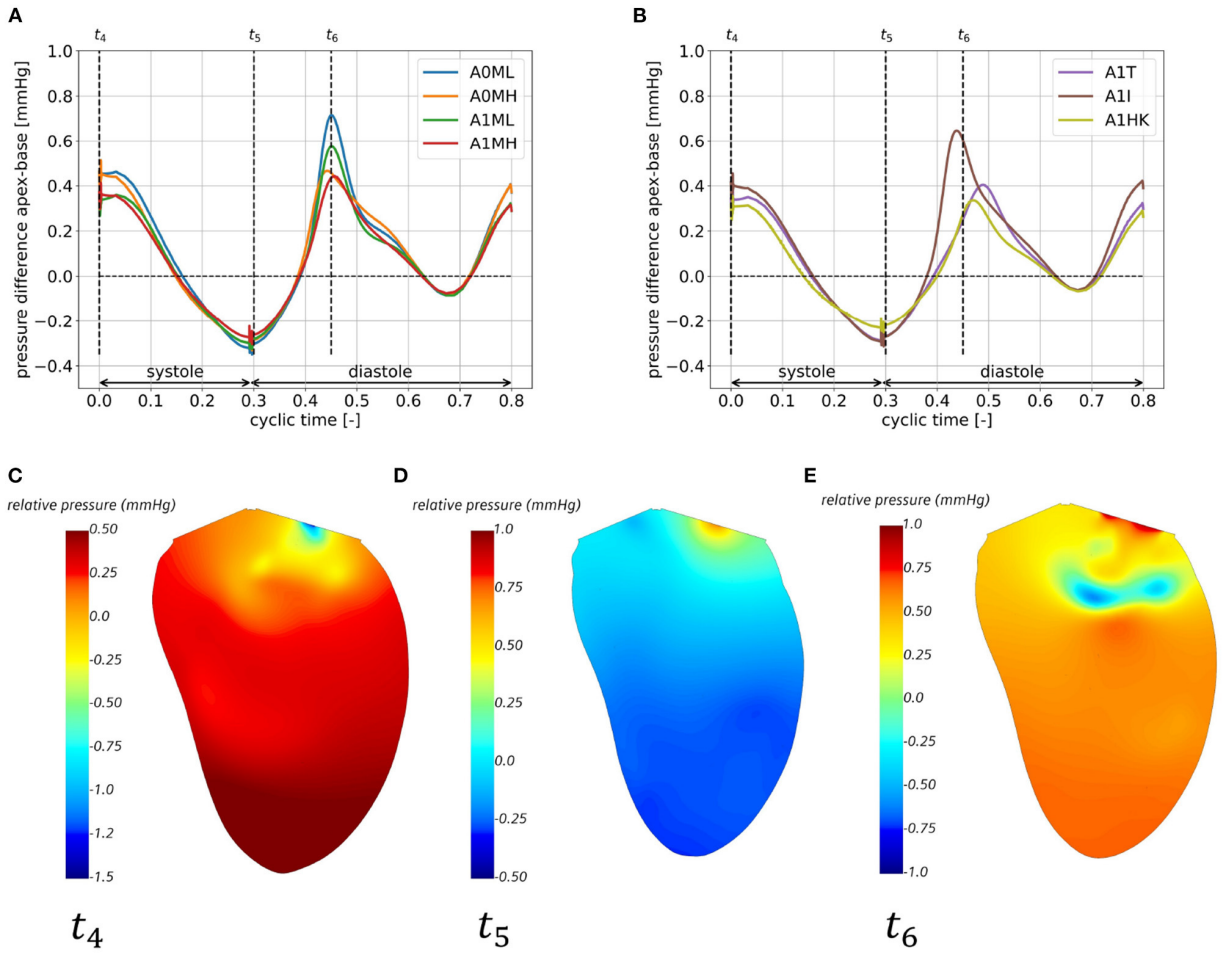
Apex-base pressure difference for cases A0ML, A0MH, A1ML, and A1MH **(A)** and cases A1T, A1I, A1HK **(B)** in the eighth cycle and relative pressure field in a half cut along the LV long axis, exemplary for case A0MH at the onset of systole at *t*_4_ **(C)**, the onset of diastole at *t*_5_ **(D)** and shortly after peak E-wave at *t*_6_ **(E)**.

Figures 10A,B show the pressure gradient from apex to base for all seven cases, whereas Figures 10C–E exemplary show the relative static pressure field in a three-chamber view of case A0MH for three representative time steps. The temporal course of the pressure gradients is similar in all cases with two maxima and two minima: the first flat maximum (Figures 10A,B at *t*_4_) at the onset of systole lasts for approximately 50 ms. The resulting intraventricular pressure gradient inside the LV accelerates blood toward the LVOT (Figure 10C). During systole, the pressure gradient decreases and achieves a minimum at the onset of diastole (Figures 10A,B,D at *t*_5_). This negative pressure gradient pointing from base to apex supports the early diastolic filling (Figure 10D). During early diastole, the pressure gradient shifts its sign a second time, reaching the second maximum after E-wave (Figures 10A,B at *t*_6_). The positive pressure gradient decelerates inflowing blood from the LA (Figure 10E). During diastasis, the pressure gradient decreases again, reaching the second minimum approximately at atrial contraction, when the A-wave is accelerated into the LV. Comparing the cases among each other, the major differences can be seen in the magnitudes of the second maximum in the pressure difference with higher values found for both cases with lower MR (A0ML and A1ML) as well as in case A1I.

## 4. Discussion

In this study, two SSMs were developed in order to describe two populations of patients after myocardial infarction: patients with and without LV aneurysms. Despite advances in drug and device therapy, heart failure subsequent to myocardial infarction remains a frequent cause of death. Thus, e.g., enhancement of therapeutic approaches that address LV remodeling following scar formation is highly desired to improve life expectancy and quality for patients with heart failure. Image-based CFD could thereby play a supportive role by modeling and predicting post-operative states. All the here considered patients are characterized by enlarged LVs, a condition which is often accompanied by MR, low EF, abnormalities in myocardial wall motion as well as myocardial wall thinning. Using these SSMs, seven mean cases are created, each representing a respective pathological configuration of MR grade and LV aneurysm. For each case, a mean end-diastolic shape is generated via the SSM together with the deformation field of the contraction from end diastole to end systole. This serves as a basis for an analysis of intraventricular hemodynamics via CFD. The numerical hemodynamic study was done using a recently proposed CCT-based CFD methodology, which was improved with respect to the earlier study in terms of physiological complexity and toward clinical translation. For a detailed discussion on the computational model, the reader is referred to Obermeier et al. (29). In order to improve the potential of the methodology toward clinical translation, the level of automation in pre-processing procedures was increased and solver settings were optimized, resulting in a 30 % reduction of the total required time for pre-processing, simulation, and post-processing. To investigate the potential of the CCT-based CFD model to reveal differences in hemodynamics between the different pathological states, various hemodynamic parameters were analyzed with the aim of identifying the best suitable biomarkers to characterize pathological features of intraventricular flow.

### 4.1. Statistical Shape Model

The two developed SSMs, which are based on multi-phase CCT data of 125 patients after myocardial infarction, aimed at providing BCs for numerical modeling of intraventricular hemodynamics. This aim was successfully realized. Additionally, the SSMs allow to analyze LV shapes by comparison of modes identified by PCA. A comparison of the first three modes showed differences in shapes and myocardial wall movement between the SSMs that are associated with presence of the LV aneurysm. The SSMs successfully represent the major features of the investigated pathology, including local shape changes due to aneurysm development and LV volume enlargement with sphericity change as well as an ability to represent hypokinetic segments caused by antero-apical, or antero-apical and basal LV aneurysms and/or scar formation. This affirms the potential ability of the SSM to be used as a diagnostic tool on its own as it was also proposed in other studies (47). However, our major aim was to describe the complex shape and contraction of the LVs in a simple way.

There is a set of earlier developed SSMs or so-called atlases for the LV (19, 47–50). Bai et al. (49) and Gilbert et al. (48) also give an overview of studies in which LV SSMs were developed. These SSMs were based on different imaging modalities including CT (19), MRI (47, 49), and echocardiography (50), which are - depending on spatial and temporal resolution - better or less suitable for subsequent use in numerical simulations. An SSM based on CCT can, however, be assumed to exhibit a robust quality regarding spatial resolution and independence of image quality from user-biases.

All of the SSMs, as well as ours, show a rather smooth ventricular shape neglecting the details of papillary muscles and trabeculae carneae. Also, the size of datasets used for SSM generation varies between approximately 100 as used in our study and several thousands. Both SSMs developed in our study describe 90 % shape variance with approximately 30 modes. Thus, the number of cases used to generate these SSMs as well as the representative cases A0ML, A0MH, and A1ML can be considered sufficient. However, the number of cases used to generate the representative cases A1MH, A1T, A1I, and A1HK seems to be low and should be increased in the future. The required number of modes to represent the shape variance is much larger than the number of modes describing the variance of normal subjects [e.g., eight in Bai et al. (49)]. A similar behavior can be noted by a SSM describing normal subjects as well as patients with dilated cardiomyopathy or with heart valve diseases, requiring 92 modes to describe 99 % shape variance (50). Beside the limited number of cases used in the development of the SSMs, the SSM methodology used in this study is limited to linear PCA. Nonlinear PCA (51) should be investigated in the future. Since SSMs simplify the shape and the contraction motion, these simplifications could be associated with uncertainties or bias in CFD results. This impact also requires to be investigated separately. Finally, it is desired in the future to extend the current SSM of the LV by shape models describing LA, ascending aorta as well as AV and MV.

Looking closer at the ventricular shape of the SSM-created mean shapes A1T, A1I, and A1HK (see Figure 6 at *t*_1_), one can see the curving and the characteristic neck resulting from myocardial thickening in case of A1T and the curving near the mid-LV anteroseptal and inferoseptal segment (segments 8 and 9) as described by Di Donato et al. (30). Regarding the mean shapes of cases A1T, A1I, and A1HK, less movement is observed in the aneurysmatic regions. We thus conclude the created mean shapes to represent the respective pathology in an adequate manner.

### 4.2. Left Ventricular Hemodynamics

The comparison of intraventricular flow features between SSM-based mean cases represents the major focus of this study. To analyze the intraventricular hemodynamics, a set of qualitative and quantitative measures were used, including large-scale flow features, energetic aspects, washout behavior as well as flow driving forces using pressure field information. These markers allow to identify differences between the investigated groups.

In an earlier study (29), we investigated four patient-specific cases each originating from one of the here presented cohorts A0ML, A0MH, A1ML, and A1MH. We found similar large-scale flow patterns in the representative and individual cases, with diastolic jets becoming less coherent with rising MR grade. This finding also correlates well with MRI-based observations (8) and is likely to be caused by the highly disturbed flow in the LA due to the regurgitation jet (29, 52). However, the individual cases showed a higher shape variability and complexity compared to the mean cases that results in higher individual differences regarding diastolic jet direction and regions of blood stagnation. Considering the impact of the MR on the intraventricular hemodynamics, we found increased systolic SED with rising MR grade in both individual and mean cases. This could be associated with an energy loss on small scales due to the regurgitation jet. This results in a lower energetic maximum of the SKE during systole and probably in a rising ratio of SKE at peak diastole to peak systole as also reported in the MRI study by Al-Wakeel et al. (8). Note, that the results associated with the regurgitation jet should be considered with caution due to a simplified modeling of the MV shape and generic valve opening and closing.

Considering differences in SKE between non-aneurysmatic and aneurysmatic cases A0ML, A0MH, A1ML, and A1MH, where SVs are comparable, the lower cyclic-mean of the aneurysmatic cases indicates a less energetic redirection of blood through these LVs, which may be caused by pathological contractions in aneurysm regions. This may lead to higher loads the LVs have to overcome to ensure a stable cardiac output and may in turn favor further remodeling (1).

Concerning the intraventricular pressure field, our results are in agreement with published pressure field distributions in a three-chamber view and the temporal course of apex-base pressure gradients (53). In the future, the pressure field analysis could be extended by an analysis of hemodynamic forces as described by Vallelonga et al. (54). Note, that the current numerical framework considers the LV as an isolated organ, thus impeding assessment of absolute pressure values. Extending the current model by a Lumped Parameter Model [e.g., a three-element Windkessel model as done by Gao et al. (55)] is a necessary next step to include an LA-LV and LV-arterial coupling.

Comparing the blood washout of the four mean cases (aneurysmatic and non-aneurysmatic with high and low MR), a better blood washout is observed in the non-aneurysmatic cases, matching clinical observations (56). As LVEDVs, SVs, and EFs of these four cases are comparable, the reason is likely to be found in the mean shapes and ventricular contraction. In the aneurysmatic cases, less movement is seen in the apex region (i.e., segments 13–17), possibly interfering with a suction effect and mixing in this area. When we investigated the four patient-specific cases in Obermeier et al. (29), we contra-intuitively found a slightly better washout in the aneurysmatic-cases. Yet, the SVs of these cases were 25–30 % higher than those of the non-aneurysmatic cases. This may indicate SSM-based mean shapes to be less affected by individual variations and provide more generalizable results. However, such a statement must be tested in depth by comparing the mean cases to a variety of patient-specific cases.

Considering the washout, focus should be attended on the apex region because this is a region of high thrombus formation risk after myocardial infarction (57–59). These clinical studies also note thrombus formation occurring preferably in patients with anterior myocardial infarction, whereas thrombus formation in patients with inferior infarction or anterior infarction without severe apical-wall-motion abnormality is rare. This correlates well with our results, showing regions with poor washout behavior (especially in the apex region) as visible in Figures 8, 9 at 0.92 s.

Interesting observations are also made when comparing the washout of different aneurysm types (i.e., cases A1T, A1I, and A1HK). While the direct flow rate of fresh blood in case A1T (30.8 %) is in the same range as observed in healthy LVs (44 ± 11 %) (60), there is still 20 % of old blood present after eight cycles, which is high even in comparison to the other pathological states. This underlines the necessity in washout analysis to evaluate fresh as well as old blood. To emphasize is furthermore the extremely poor washout in case A1HK, in terms of fresh and old blood. A connection to the contraction field seems likely. In this case, less movement in basal and mid LV regions is visible, whereas the strongest movement is present in the apex. When viewing the pathway of blood (Figure 9) of case A1HK, movement and mixing can primarily be observed in longitudinal direction toward the apex, whereas mixing in radial direction is small. From these observations, a hypokinetic movement can be linked to an increased risk of thrombus formation in comparison to other aneurysm types.

Summing up, the SSM-created mean cases are able to represent characteristic flow features that were previously observed in patient-specific investigations of the respective pathology (higher systolic SED and less coherent diastolic jets in MR) and are in line with clinical observations (worse washout in aneurysmatic cases). Our findings show differences in intraventricular hemodynamics associated with different LV remodeling changes caused by myocardial infarction: local (aneurysm development) and global (volume and sphericity index increase) changes in shape as well as development of an MR of varying severity and abnormalities of the myocardial wall movement with hypokinetic segments. In combination with further model developments and investigations toward the selection of best suitable hemodynamic biomarkers assessing heart illnesses, the proposed numerical framework may support treatment decisions by distinguishing different pathological states from healthy states and among each other.

### 4.3. Model Discussion and Limitations

Modeling the complex LV anatomy and physiology in a way that points toward translation into clinical routine induces some simplifications, which are emphasized in the following.

The incorporation of valves with their complex shape and motion poses a major challenge in a detailed numerical modeling of the LV (39, 61). We modeled the valves in 2D via a porous baffle interface. This is a simplified approach, which delivered reasonable results in recent studies (38, 39). In our study, the 2D valve representation induced the formation of ring vortices below the MV during both E-wave and A-wave, as visible in the visualization of Q-criterion isosurfaces provided as Supplementary Material. These ring vortices were observed, e.g., in Ebbers et al. (53) and discussed in detail by Pedrizzetti et al. (1). In the Q-criterion visualizations it furthermore becomes visible, that with rising MR grade, vortex decay in diastole starts earlier, an observation that correlates well with the study of Al-Wakeel et al. (8).

In the MR-cases, a generic planar regurgitation area is applied, such that a reasonable pressure loss over the MV results and the case-specific MR grade (regurgitation volume in % of the SV) is met. Following this approach, the 3D character of the valves cannot be accounted for and the projected orifice area in diastole as well as during MR cannot be realized in a case-specific manner. This also includes the modeling of a regurgitation jet angle, which could not be obtained from our data base. As the here investigated cases are SSM-based and non-patient-specific, including these characteristics would, e.g., require an additional SSM per valve. As the valves and their movement could not be accurately captured in the available CCT data, this was not possible at current development state. The same applies for case-specific modeling of LA, aorta, papillary muscles, and trabeculae. Extending the model by these aspects is planned in future development steps. For a detailed discussion on the impact of these simplifications onto the intraventricular hemodynamics, the reader is referred to Obermeier et al. (29).

The LV motion is derived based on end-diastolic and end-systolic states. Temporal resolution of the CCT data did not allow for an accurate tracking of intermediate states. Consequently, phenomena as the torsional motion of the LV cannot be implemented. During systole, apex and base are rotating in opposite directions. This twisting mechanism is associated with a momentum change of blood, which may affect intraventricular flow patterns (62). Yet, the influence of LV torsional motion onto flow and energy dynamics was investigated via image-based CFD by Vasudevan et al. (63) and concluded to be insignificant. Similar results are reported from the image-based CFD model of Canè et al. (64), where torsional motion did not influence energy loss and only slightly affected velocity, vorticity and wall shear stress. Our approach of two-state-based deformation derivation is motivated by a minimization of data requirements of the computational model in the sense of a possible translation of the model to clinical routine workflows. Yet, the influence of neglecting intermediate states remains to be clarified in depth, e.g., via a study on the impact of different deformations on flow parameters like inflow orientation and vortex formation.

Last to discuss is the incorporated model for small scale flow features. In the context of turbulence analysis, a Large Eddy Simulation (LES) is more appropriate than RANS, due to the transitional flow regime (65, 66). Yet, the present study does not focus turbulence on analysis. Further, the increasing requirements on the computational mesh as well as the need to compute a significantly larger number of cells when using LES does not align with the goal of a development toward clinical translation. Furthermore, in the context of ventricular assist devices, Zhang et al. (67) compared several turbulence models to experimental data, and all models reasonably replicated the fluid flow. A study opposing RANS and LES models in LV hemodynamics might, however, provide further insights.

## 5. Conclusion

The proposed CCT-based analysis of hemodynamics combined with SSM-based description of the heart shape and contraction pattern for subcohorts of patients seems to be a promising approach facilitating an analysis of intracardiac flow. Modeling of hemodynamics of mean shapes as a kind of reduced order modeling instead of patient-specific simulations could accelerate intracardiac flow analysis and reduce requirements in computational power that is necessary to translate the modeling-based analysis of intraventricular flow into the clinic. The proposed approach has the potential to increase the significance of CCT for diagnosis and treatment decisions. However, further enhancement of the computational framework, identification of suitable hemodynamic parameters as well as a clarification of the reduced order modeling level and its representativeness are necessary to embed the approach in clinical routine workflows to support clinicians.

## Data Availability Statement

The data sets (geometries and applied boundary conditions) presented in this study are provided as open data in an online repository. The name of the repository and accession number can be found at: doi: 10.6084/m9.figshare.19328687.

## Ethics Statement

The studies involving human participants were reviewed and approved by the Ethics Committee - Charité Universitätsmedizin Berlin (EA2/177/20). The study was performed according to the principles of the Declaration of Helsinki.

## Author Contributions

LG, NS, CK, and TK: conceptualization. LO, ASz, NS, ON, and CK: data curation. LO, LT, KV, JB, HL, and ASc: formal analysis and methodology. LG and TK: funding acquisition and supervision. LO and ASc: visualization. LO, NS, ON, CK, and LG: investigation. LO, KV, ASz, LG, and NS: writing original draft. All authors: review and editing. All authors contributed to the article and approved the submitted version.

## Funding

This study was funded by the Einstein Center Digital Future and by the DFG grants for the project Nr. 465178743 in frames of the SPP2311.

## Conflict of Interest

HL and ASz were employed by 1000shapes GmbH, which received funds from Charité-Universitätsmedizin Berlin to create the statistical shape model and perform the presented research in relation to the statistical shape model. The remaining authors declare that the research was conducted in the absence of any commercial or financial relationships that could be construed as a potential conflict of interest.

## Publisher's Note

All claims expressed in this article are solely those of the authors and do not necessarily represent those of their affiliated organizations, or those of the publisher, the editors and the reviewers. Any product that may be evaluated in this article, or claim that may be made by its manufacturer, is not guaranteed or endorsed by the publisher.

## Supplementary Material

Visualizations of Q-criterion isosurfaces of all cases throughout the cardiac cycle are provided as Supplementary Material and can be found online at: https://www.frontiersin.org/articles/10.3389/fcvm.2022.901902/full#supplementary-material
