# Supplementary material for: CT-Based Analysis of Left Ventricular Hemodynamics Using Statistical Shape Modeling and Computational Fluid Dynamics
Source: Front Cardiovasc Med. 2022 Jul 5;9:901902. doi: 10.3389/fcvm.2022.901902 (PMC9294248; doi:10.3389/fcvm.2022.901902)
Supplement: Supplementary file 1 [file Data_Sheet_1.PDF]

Supplementary Material

1 VORTEX VISUALIZATION

Figures S1 and S2 visualize vortex structures of the seven mean cases via isosurfaces of the Q-criterion throughout the cardiac cycle. In all cases, vortical flow regurgitates into the LA in systole at t_1. In

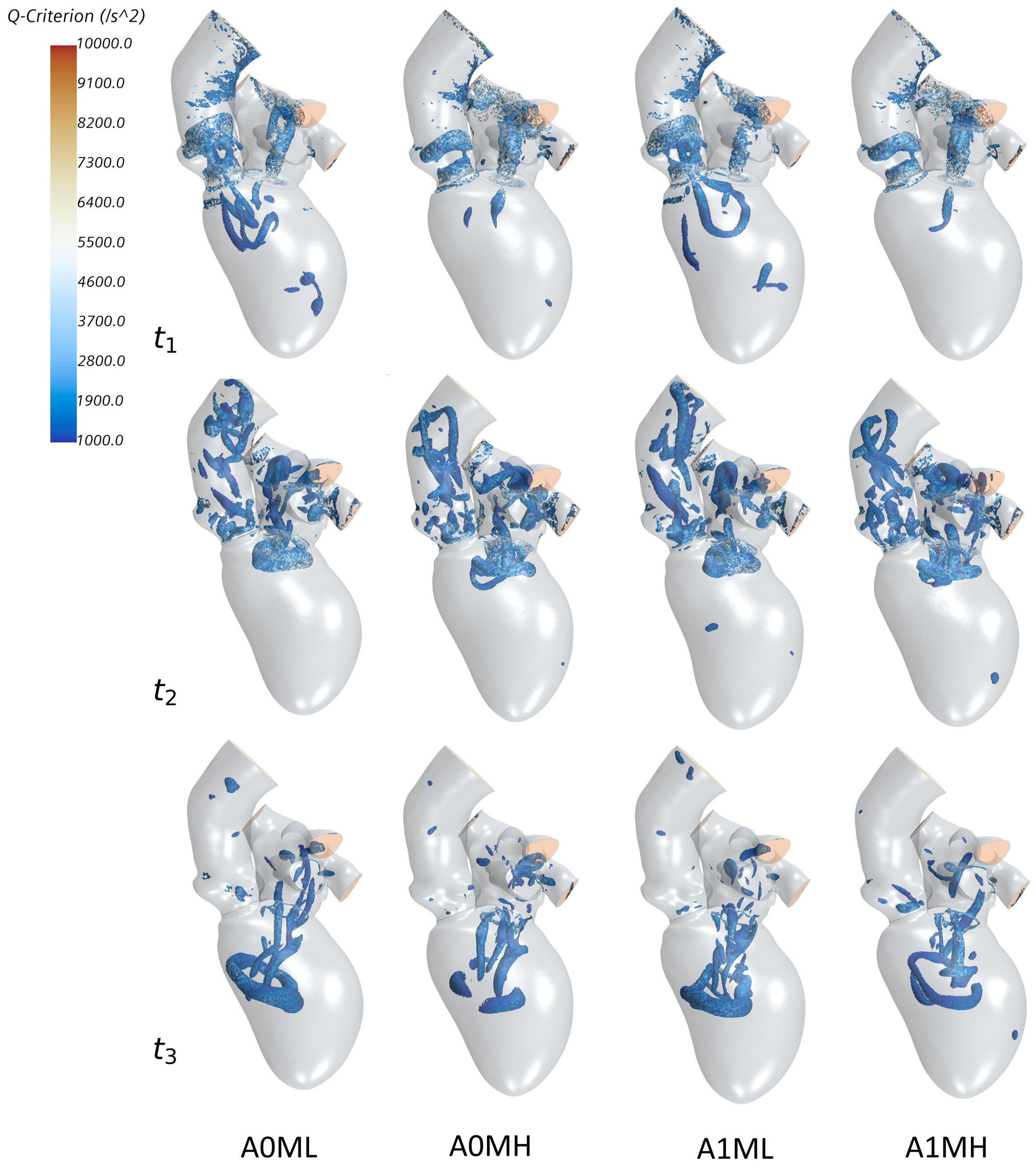

Figure S1. Q-criterion isosurfaces of cases A0ML, A0MH, A1ML, and A1MH from left to right at t_1: peak systole (top), t_2: peak E-wave (mid), and t_3: diastasis (bottom) of the seventh cycle.

early diastole (t_2), ring vortices form below the MV and accompany the E-wave jet towards the apex, disintegrating in the temporal course of diastole. Only in the low MR cases (i.e., case A0ML, and A1ML), a coherent ring structure is still visible at diastasis (t_3). At atrial contraction, a second ring vortex forms below the MV in all cases.

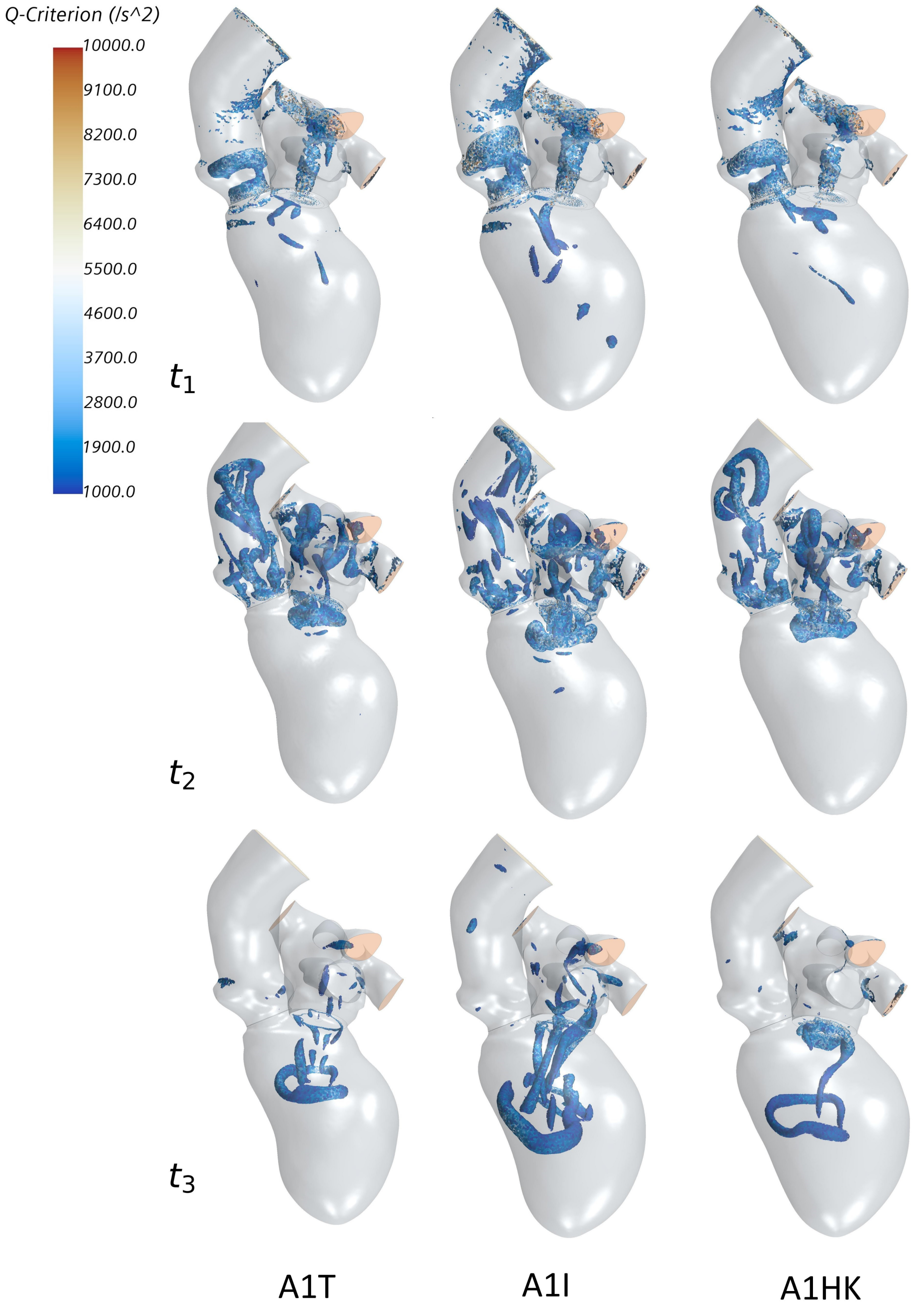

Figure S2. Q-criterion isosurfaces of cases A1T, A1I, and A1HK from left to right at t_1: peak systole (top), t_2: peak E-wave (mid), and t_3: diastasis (bottom) of the seventh cycle.
